# Topical Therapeutic Efficacy of Ebselen Against Multidrug-Resistant *Staphylococcus aureus* LT-1 Targeting Thioredoxin Reductase

**DOI:** 10.3389/fmicb.2019.03016

**Published:** 2020-01-15

**Authors:** Chuanjiang Dong, Jingxuan Zhou, Peng Wang, Tao Li, Ying Zhao, Xiaoyuan Ren, Jun Lu, Jun Wang, Arne Holmgren, Lili Zou

**Affiliations:** ^1^The First College of Clinical Medical Science, China Three Gorges University, Yichang, China; ^2^The Institute of Infection and Inflammation, Medical College, China Three Gorges University, Yichang, China; ^3^Key Laboratory of Luminescent and Real-Time Analytical Chemistry (Southwest University), Ministry of Education, College of Pharmaceutical Sciences, Southwest University, Chongqing, China; ^4^Division of Biochemistry, Department of Medical Biochemistry and Biophysics, Karolinska Institutet, Stockholm, Sweden; ^5^Translational Neuroscience & Neural Regeneration and Repair Institute/Institute of Cell Therapy, The People’s Hospital of China Three Gorges University, Yichang, China

**Keywords:** ebselen, thioredoxin reductase, topical treatment, *Staphylococcus aureus*, curcumin

## Abstract

As a thiol-dependent enzyme, thioredoxin reductase (TrxR) is a promising antibacterial drug target. Ebselen, an organo-selenium with well-characterized toxicology and pharmacology, was recently reported to have potent antibacterial activity against *Staphylococcus aureus.* In this paper, we demonstrated that ebselen has strong bactericidal activity against multidrug-resistant (MDR) *S. aureus* based on taking TrxR as a major target and disruption of the redox microenvironment. Further, the topical therapeutic efficacy of ebselen for staphylococcal skin infections was assessed in a rat model. Treatment with ebselen significantly reduced the bacterial load and the expression of pro-inflammatory cytokines tumor necrosis factor-α (TNF-α), interleukin-6 (IL-6) and interleukin-1 beta (IL-1β) in *S. aureus* skin lesions; further, wound healing and pathological changes were obvious improved in ebselen-treated rats compare to controls. Finally, ebselen was found to sensitize *S. aureus* to curcumin, which may be due to their synergistic effects in inhibiting bacterial TrxR. Altogether, ebselen is an effective topical antibacterial agent in animal model of MDR *S. aureus* LT-1 skin infection. This may lay the foundation for further analysis and development of ebselen as an antibacterial agent for topical treatment of MDR staphylococcal infections.

## Introduction

Novel antimicrobials and new bacterial cell targets are urgently needed to overcome the ever-increasing worldwide antimicrobial resistance (AMR), which is now widely accepted as a global health threat with leading causes of morbidity and mortality (Brown and Wright, 2016; Baptista et al., 2018). In May 2018, the World Health Organization (WHO), the Food and Agriculture Organization of the United Nations (FAO), and the World Organization for Animal Health (OIE) signed a Memorandum of Understanding (MoU) to support governments, health care workers, and stakeholders to engage and collaborate in the fight against AMR (Balkhy et al., 2018). Limiting the emergence of AMR is critical to preserve our ability to treat bacterial and microbial infections not only in humans, but also in animal, plant, food, and environment circumstances (Bhatia, 2018).

*Staphylococcus aureus* is a highly adaptable, “Janus-faced” Gram-positive pathogen, for which humans are the only known reservoir (Kobayashi et al., 2015; Balasubramanian et al., 2017; Dweba et al., 2018). *S. aureus* is present in approximately 30% of the human population, and its presence has been linked to skin rashes, wound infections, pleuropulmonary, bacteremia, infective endocarditis, and device-related infections (Coates et al., 2014; Tong et al., 2015). In the pre-antibiotic era, the case fatality rate (CFR) for *S. aureus* was ∼80%; it has since decreased and plateaued at 15∼50% over the past several decades since the introduction of penicillin (van Hal et al., 2012). However, the adaptive evolution of *S. aureus* during the modern antibiotic era has enabled its acquisition of antibiotic resistance, thus increasing disease burden worldwide (Pantosti et al., 2007; McGuinness et al., 2017).

**FIGURE 1:**
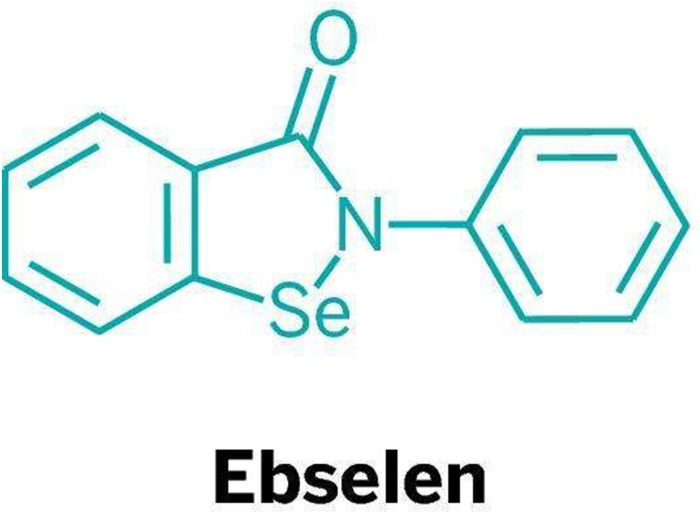
Chemical structure of ebselen.

Ebselen or 2-phenyl-1,2 benzisoselenazol-3(2H)-one, an organo-selenium compound, is a clinical trial drug with well-characterized toxicology and pharmacology (Zou et al., 2017; Figure 1). Recent studies have shown that ebselen possesses bactericidal activity against Gram-positive, including multidrug-resistant (MDR) clinical isolates of *S. aureus*. Bacterial thioredoxin reductase (TrxR) was assessed to be a critical target, and the ebselen inhibition is through a reaction with the active site dithiol of TrxR to act as a competitive inhibitor (Lu et al., 2013; Thangamani et al., 2015; Zou et al., 2018). Bacterial TrxR transfers an electron from NADPH to Trx allowing it to regulate functions of various critical cellular proteins, including ribonucleotide reductase (RNR), methionine-S-sulfoxide reductase (Msr), thiol peroxidase (Tpx), bacterioferritin comigratory protein (Bcp) (Holmgren, 1985; Lu and Holmgren, 2012, 2014a; Leitsch, 2017). Bcp is critical in antioxidant defense and DNA synthesis and repair (Lu and Holmgren, 2012, 2014b). However, animal experiments testing antibacterial activity of ebselen are rare. Thus, the aim of this study was to evaluate the bactericidal activity of ebselen against MDR *S. aureus in vitro*, and to assess its topical therapeutic efficacy, anti-inflammatory properties, and potential clinical applications in staphylococcal skin infection rat model.

## Results

### Antibacterial Activity of Ebselen Targeting *S. aureus* TrxR

**FIGURE 2:**
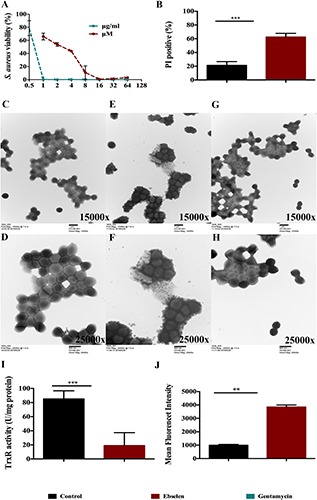
Antibacterial effect of ebselen on *Staphylococcus aureus* through targeting bacterial TrxR. *S. aureus* LT-1 cells grown to DO 600 nm of 0.4 and diluted 100 times were treated with serial dilution of ebselen, and gentamycin was used as positive control. **(A)** Antibacterial effect of ebselen on the growth of *S. aureus*. Bacterial growth was presented by measuring OD_600 nm_. *S. aureus* LT-1 cells grown to DO 600 nm of 0.4 and were treated with 22 μg/ml ebselen. **(B)** Mean ± SD of propidium iodide (PI)-stained *S. aureus* LT-1 by Flow cytometry; **(C–H)** Transmission electron microscopy of *S. aureus* treated with ebselen; **(C,D)** control; **(E,F)** 22 μg/ml ebselen; **(G,H)** 64 μg/ml gentamycin; **(C,E,G)** 15000x; **(D,F,H)** 25000x; **(I)** TrxR activity was assayed for DTNB reduction in the presence of Trx in *S. aureus* LT-1 extracts; **(J)** Mean fluorescent intensity (MFI) Means ±SD of H_2_DCF-DA-stained *S. aureus* LT-1 were detected to present ROS level. (^∗∗^*P* < 0.01; ^∗∗∗^*P* < 0.001; student’s *t*-test. Data are presented as means ±SD of three independent experiments).

*Staphylococcus aureus* LT-1 was isolated from patients with cutaneous infections in The first clinical hospital of Yichang (China), and identified as an MDR strain (Tables 1, 2 and Supplementary Figure S1). LT-1 cells with logarithmic growth were treated with different concentrations of ebselen for 16 h. The antibacterial effect of ebselen on the growth of *S. aureus* was investigated in microplates by a spectrophotometer, which estimated cell number. As shown in Figure 2A, ebselen inhibited *S. aureus* growth with a minimal inhibition concentration (MIC) of 2.2 μg/ml (8 μM). Meanwhile, the positive control gentamycin inhibited *S. aureus* growth with a MIC of 0.85 μg/ml (1.08 μM). Further, the propidium iodide (PI) nuclear staining which represents the bacterial membrane permeability was performed after treatment with 22 μg/ml ebselen. PI stains the nucleic acids inside dead cells, or those with damaged membranes. In agreement with the inhibitory effect on bacterial growth curve, when LT-1 cells were treated with ebselen, there was a significant increase in PI positive cells (*P* < 0.001, Figure 2B).

**TABLE 1:** Biochemistry identification of clinical isolated *Staphylococcus aureus* LT-1.

2 AMY	–	16 BGAR	–	28 AlaA	–	44 NAG	+	57 dRAF	–
4 PIPLC	–	17 AMAN	–	29 TyrA	–	45 dMAL	+	58 O129R	+
5 DXY	–	19 dSOR	–	30 dSOR	+	46 BACI	+	59 SAL	–
8 ADH1	+	20 LeuA	–	31 URE	+	47 NOVO	–	60 SAC	+
9 BGAL	+	23 PROA	–	32 POLYB	–	50 NC6.5	+	62 dTRE	+
11AGLU	+	24 BGURr	–	37 dGAL	+	52 dMAN	+	63 ADH2s	+
13APPA	–	25 AGAL	–	38 dRIB	+	53 dMNE	+	64 OPTO	+
14CDEX	–	26 PyrA	+	39 ILATK	+	54 MBdG	+		
15 AspA	–	27 BGUR	–	42 LAC	–	56 PUL	–		

*AMY, amygdalin; PIPLC, phosphatidyl phosphatase C; dXYL, D-xylose; ADH1, arginine double hydrolase 1; BGAL, β-D-galactosidase; AGLU, α-glucosidase; APPA, alanine- phenylalanine-proline aromaminase; CDEX, cyclodextrin; AspA, L-aspartate arylamine; BGAR, β-galactopyranosidase; AMAN, α-Mannosidase; PHOS, phosphatase; LeuA, leucine aromaminase; ProA, proline aromaminase; BGURr, β-glucuronidase; AGAL, α-galactosidase; PyrA, pyroglutaminase; BGUR, β-D-glucuronidase; AlaA, alanine aromatase; TyrA, tyrosine aromaminase; dSOR, D-sorbitol; URE, urease; POLYB, polycolistin B tolerance; dGAL, D-galactose; dRIB, D-ribose; lLATk, lactate produces alkali; LAC, lactose; NAG, N-acetyl-D-glucosamine dMAL D-Maltose; BACI, bacillus peptide tolerance; NOVO, novomycin tolerance; NC6.5, 6.5% NaCl growth; dMAN, D-mannitol; dMNE, D-mannose; MBdG, methyl-B-D- glucopyranoside; PUL, pullulan; dRAF, D-raffinose; O129R, O/129 tolerant; SAL, salicin; SAC, saccharose; dTRE, D-trehalose; ADH2s, arginine double hydrolase 2; OPTO, optoxin tolerance.*

**TABLE 2:** Antimicrobial susceptibility test of *S. aureus* LT-1.

**Antibiotics**	**Diameter (mm)**	**Cut-off**	**Sensitivity/resistance**
Gentamicin	23	12–15	(S)
Levofloxacin	28	15–19	(S)
Ciprofloxacin	24	15–21	(S)
Selectin	30	10–16	(S)
Tetracycline	14	14–19	(R)
Penicillin	10	28–29	(R)
Oxacillin	6	10–13	(R)
Vancomycin	0.38	2–16	(S)
Erythromycin	6	13–23	(R)
Clindamycin	6	14–21	(R)
Rifampicin	31	16–20	(S)
Linezolid	31	20–21	(S)
Chloromycetin	26	12–18	(S)

The effect of ebselen on the morphology of *S. aureus* was detected by transmission electron microscopy (Figures 2C–H). The morphology of *S. aureus* changed significantly when treated with ebselen compared to the control. Untreated *S. aureus* cells have a smooth surface and a complete cell membrane and cell wall (Figures 2C,D). After 20 min treatment with 22 μg/ml ebselen, the *S. aureus* cell membrane and cell wall were ruptured, cytoplasmic material flowed out, and the cells eventually died (Figures 2E,F). In contrast, gentamycin-treated *S. aureus* cells showed no obvious morphological changes compared to untreated cells (Figures 2G,H).

The inhibitory effect of ebselen on *S. aureus* TrxR activity was detected by dithiobis nitrobenzoic acid (DTNB) assay. Interestingly, ebselen treatment significantly inhibited bacterial TrxR activity when compared with untreated cells (*P* < 0.001, Figure 2I). In addition, the mean fluorescent intensity (MFI) of reactive oxygen species (ROS) was detected by Flow cytometry, and showed that the ROS production level in ebselen-treated *S. aureus* cells was significantly upregulated compared to the control (*P* < 0.01, Figure 2J).

Altogether, these results shown that ebselen is an effective antibacterial compound that inhibits *S. aureus* TrxR *in vitro*, and that ROS production is one of the key virulent factors for its bactericidal activity.

### The Therapeutic Efficacy of Ebselen in Rat Model of Staphylococcal Skin Infections

**FIGURE 3:**
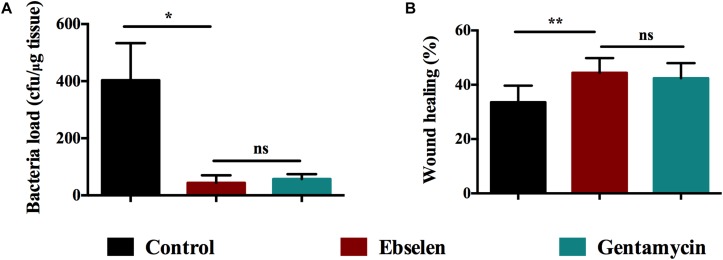
Therapeutic efficacy of ebselen in treating staphylococcal skin lesions. Rats were treated with 25 mg/Kg ebselen, 5 mg/Kg gentamycin (positive control) and PBS once daily for 5 days, respectively. **(A)** The bacterial load was calculated by counting the colonies; **(B)** The wound healing was evaluated according to the formula as follows: *F* = (A-B)/A^∗^100%, where F: wound healing rate, A: original wound area, B: wound area post-treatment. (^∗^*P* < 0.05; ^∗∗^*P* < 0.01; student’s *t*-test. Data are presented as means ±SD of three independent experiments).

Thirty rats were randomly divided into three groups: control group, ebselen-treated group, and gentamycin-treated group. Thick scalpel cuts were made into the dermis of the skin on the posterior upper back and neck of rats and inoculated with *S. aureus* LT-1 cells. Two days post-infection, the bacteria from the three groups showed no obvious difference in colony forming units (average of 3.8 ± 1.0 (Log_10_^3^) CFU/ml) (*P* < 0.05). Rats were then treated topically with 25 mg/kg ebselen, 5 mg/kg gentamycin or PBS once per day for 5 days, respectively. The results are highlighted in Figure 3A. After 5 days post-treatment, the rats receiving ebselen had a significantly reduced mean bacterial count compared with the group receiving PBS (*P* < 0.05). The ebselen-treated group had the highest reduction (43 ± 26.4 CFU/ml), followed by gentamycin (57 ± 17.3 CFU/ml) and control (403 ± 130.1 CFU/ml). There was no obvious difference between ebselen and gentamycin in their antibacterial activities (*P* > 0.05).

Wound healing was also evaluated according to the formula as follows: *F* = (A−B)/A^∗^100%, where F: wound healing rate, A: original wound area, and B: wound area post-treatment. As the results showed in Figure 3B, ebselen-treated rats had a wound healing rate that was significantly higher than the control (*P* < 0.01). The group treated with ebselen had the highest healing rate in wound (44.43 ± 5.41), followed by gentamycin (42.43 ± 5.54), and PBS (33.56 ± 6.12). There was no difference in wound healing in rats treated with ebselen and gentamycin (*P* > 0.05).

### Topical Effect of Ebselen on Inflammatory Cytokines of Staphylococcal Skin Infection

**FIGURE 4:**
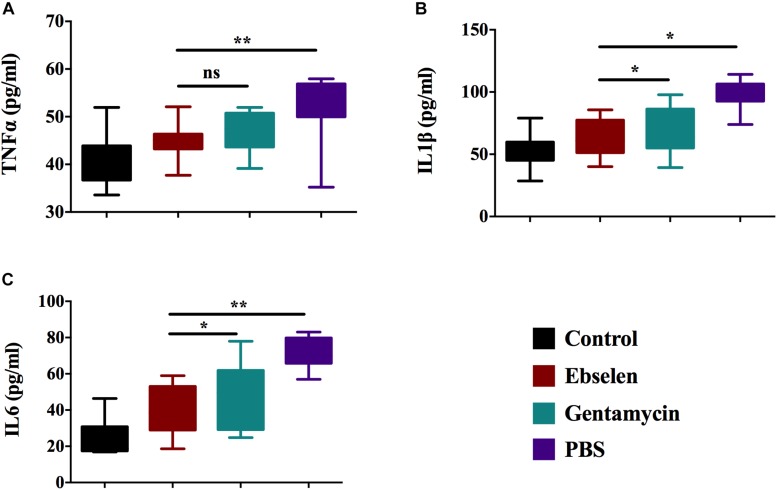
Efficacy of ebselen on cytokines production in staphylococcal skin lesions. Blood serum from each rat was used for cytokine detection by ELISA. **(A)** Tumor necrosis factor-α (TNF-α); **(B)** interleukin-6 (IL-6); **(C)** Interleukin-1 beta (IL-1β). (^∗^*P* < 0.05; ^∗∗^*P* < 0.01; student’s *t*-test. Data are presented as means ±SD of three independent experiments).

To study the immune-modulatory activity of ebselen in a topical application against *S. aureus* LT-1 skin infection, forty rats were randomly divided into 4 groups: A to D. Scalpel cuts were made in the dermis of the skin on the posterior upper back and neck of the rats. Rats from group A served as a control group and were not infected or treated. Rats from groups B to D were inoculated with the *S. aureus* strain LT-1 and further topically treated with 25 mg/kg ebselen, 5 mg/kg gentamycin, or PBS once per day for 5 days, respectively. An ELISA assay was used to measure the presence of pro-inflammatory cytokines, including tumor necrosis factor-α (TNF-α), interleukin-6 (IL-6), and interleukin-1 beta (IL-1β). As shown in Figure 4, ebselen significantly reduced the expression of all three tested pro-inflammatory cytokines compared to rats treated with PBS (*P* < 0.05). For TNF-α, the group treated with ebselen had the highest reduction in expression (44.83 ± 3.59 pg/ml), followed by gentamycin (47.01 ± 4.19 pg/ml), and PBS (51.90 ± 6.98 pg/ml) (Figure 4A), while there was no difference between ebselen and gentamycin (*P* > 0.05). For IL-1β, the group treated with ebselen had the highest reduction in expression (64.83 ± 8.91 pg/ml), followed by gentamycin (75.61 ± 7.37 pg/ml), and PBS (102.02 ± 11.22 pg/ml) (Figure 4B), while there was difference between ebselen and gentamycin (*P* < 0.05). Finally, for IL-6, the group treated with ebselen had the highest reduction in expression (38.03 ± 5.85 pg/ml), followed by gentamycin (45.21 ± 8.7 pg/ml), and PBS (71.92 ± 10.0 pg/ml) (Figure 4C), while there was difference between ebselen and gentamycin (*P* < 0.05). Overall, ebselen treatment had the greatest effect in reducing the expression of pro-inflammatory cytokines, and its anti-inflammatory activity was considerably higher than gentamycin.

**FIGURE 5:**
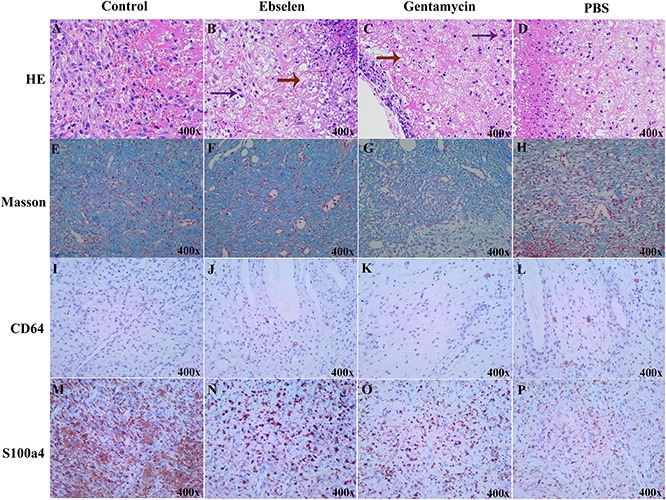
Histological results of ebselen-treated staphylococcal skin lesions. The new formative tissues from staphylococcal skin lesions rats with different treatments were used for pathological detection. **(A–D)** HE staining, red arrow indicated granulation tissues, purple arrow indicated fibroblasts; **(E–H)** Masson staining, blue color indicated collagenous fibers; **(I–L)** IHC using CD64, brown dots indicated CD64 positive cells; **(M–P)** IHC using S100a4, brown dots indicated S100a4 positive cells; **(A,E,I,M)** Control; **(B,F,J,N)** Ebselen; **(C,G,K,O)** Gentamycin; **(D,H,L,P)** PBS.

Further, hematoxylin and eosin (H&E) and Masson staining were performed using the new formative tissues. Staining revealed that PBS-treated rats had reduced number of fibroblasts and microvascular, and increased number of inflammatory cells and erythrocytes. Meanwhile, the ebselen or gentamycin-treated rats had dense granulation tissues and microvascular, and less scattered lymphocytes (Figures 5A–H). Anti-CD64 antibody was further used to detect activated mature neutrophil by Immunohistochemistry (IHC). Rats treated with ebselen had less infiltrative neutrophils than those treated with PBS (Figures 5I–L). Moreover, fibroblasts were detected by S100a4 antibody, also termed FSP-1 (fibroblast-specific protein-1). The results showed an increased number of fibroblasts in ebselen or gentamycin-treated rats compared to the PBS-treated group (Figures 5M–P).

Altogether, these results demonstrated that ebselen may influence the repair of damaged skin, and its efficacy to do so is considerably higher than that of gentamycin.

### Synergistic Activity of Ebselen With Curcumin Against *S. aureus in vitro*

As an important constituent of turmeric, curcumin, has various biological activities due to its antioxidant mechanism. Previous studies have shown that curcumin has antibacterial activity against various bacteria, including *S. aureus* (Song et al., 2012), and that it operates through blocking the assembly dynamics of filamentous temperature-sensitive protein Z (FtsZ) in the Z ring at the site of division in bacterial cells (Rai et al., 2008; Kaur et al., 2010), affecting biofilm initiation (Rudrappa and Bais, 2008). It may also have bacterial membrane lysing properties (Tyagi et al., 2015), yet whether it targets bacterial TrxR remains unknown.

**FIGURE 6:**
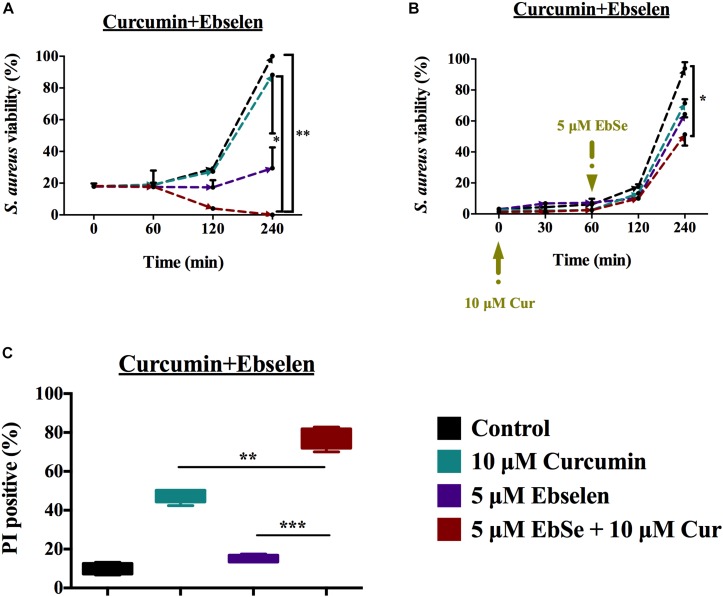
Synergistic antibacterial effect of ebselen and curcumin on the growth of *S. aureus*. *S. aureus* ATCC25923 grown to DO 600 nm of 0.4 and diluted 100 times were treated with serial concentrations of ebselen and curcumin in combination. **(A,B)** Antibacterial effect of ebselen and curcumin on the growth of *S. aureus*, and cell viability was presented by measuring OD_600__ nm_, **(A)** Ebselen and curcumin were added at the same time in the very beginning; **(B)** Curcumin was added at the very beginning, and ebselen was added 60 min later. *S. aureus* ATCC25923 grown to DO 600 nm of 0.4 were treated with 5 μM ebselen and 80 μM curcumin in combination. **(C)** Mean ±SD of propidium iodide (PI)-stained *S. aureus* by Flow cytometry. (^∗^*P* < 0.05; ^∗∗^*P* < 0.01; ^∗∗∗^*P* < 0.001; student’s *t*-test. Data are presented as means ±SD of three independent experiments).

The synergistic inhibition effect of bacterial growth by ebselen and curcumin was tested on *S. aureus* ATCC25923. The growth of *S. aureus* was substantially inhibited by 5 μM ebselen and 10 μM curcumin in 96 well-plate 4 h post-incubation (*P* < 0.01, Figure 6A). Furthermore, as showed in Figure 5B, adding 5 μM ebselen to 10 μM curcumin-treated *S. aureus* could also significantly inhibit cells growth (*P* < 0.05, Figure 6B). In addition, the Bliss model of synergism against *S. aureus* was used, and the degree of synergy of 5 μM ebselen and 10 μM curcumin is 0.89 2 h post-incubation (Supplementary Figure S2).

To detect whether treatment with 5 μM ebselen and 80 μM curcumin affected bacterial membrane permeability, PI nuclear staining was performed. Consistent with the inhibitory effect on bacterial growth, treatment with ebselen and curcumin increased the number of PI positive *S. aureus* cells compared to that of ebselen or curcumin alone (*P* < 0.01, Figure 6C).

### The Inhibition Activity of Bacterial TrxR by Ebselen and Curcumin

**FIGURE 7:**
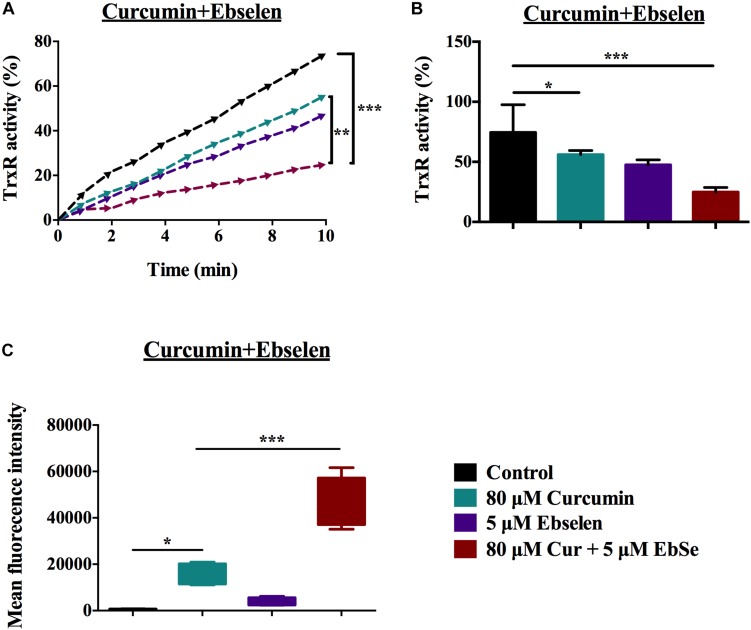
The inhibition activity of TrxR in ebselen and curcumin-treated *S. aureus*. *S. aureus* ATCC25923 grown to DO 600 nm of 0.4 were treated with 5 μM ebselen and 80 μM curcumin in combination. **(A,B)** TrxR activity was assayed using DTNB reduction in the presence of Trx in *S. aureus* extracts; **(A)** Slope curve; **(B)** Means ±SD; **(C)** Mean fluorescent intensity (MFI) of Means ± SD of H_2_DCF-DA-stained *S. aureus* were detected to present ROS level. (^∗^*P* < 0.05; ^∗∗^*P* < 0.01; ^∗∗∗^*P* < 0.001; student’s *t*-test. Data are presented as means ±SD of three independent experiments).

Thioredoxin reductase activity in *S. aureus* was assessed following treatment with 5 μM ebselen and 80 μM curcumin. Although curcumin alone could inhibit TrxR activity, the combination with ebselen exhibited a significantly stronger inhibitory effect (*P* < 0.01) (Figures 7A,B).

Since the Trx system is the major mediator in redox balance, ROS production was assessed following treatment with 5 μM ebselen and 80 μM curcumin as detected with H_2_DCF-DA by flow cytometry. When *S. aureus* cells were treated with curcumin alone, ROS production was increased (*P* < 0.05); however, the effect of ebselen and curcumin in combination on ROS production was significantly higher (*P* < 0.001) (Figure 7C).

Altogether, the above results demonstrated that targeting *S. aureus* TrxR is one of the antibacterial mechanisms of curcumin, and ebselen enhances its efficacy.

## Discussion

The WHO stated that 1 in 10 patients suffer an infection while receiving medical care and that more than half of surgical site infections may be antibiotic-resistant (Odell, 2010). Meanwhile, skin infections caused by MDR *S. aureus* have been defined as a major public health threat worldwide (McCaig et al., 2006). Therefore, new and effective treatment strategies are warranted (Antonelou et al., 2011).

As a major thiol-dependent enzyme system exists in both mammalian and most Gram-positive bacteria, Trx system transfers electrons from NADPH to corresponding substrates via TrxR, which is critical for DNA synthesis, defense against oxidative stress, repair of oxidized proteins, and post-translational modifications (Lillig and Holmgren, 2007; Tejman-Yarden et al., 2013; Leitsch, 2017; Zou et al., 2017, 2018). Bacterial TrxR has notable differences in components, enzyme structure, and reaction mechanisms compared with corresponding mammalian host (Holmgren, 1989; Sandalova et al., 2001; Lu et al., 2013), which guaranteed it act as an appropriate target by specific antibiotics. We and other groups previously found that the selenazol drug ebselen was a lead compound for this antibiotic principle (Winterbourn and Hampton, 2008; Lu and Holmgren, 2014a; Thangamani et al., 2015). Briefly, mammalian TrxR is a large (55 kDa/subunit) selenium-containing enzyme, and ebselen react with it to form Se-Se bond to be a substrate for better redox homeostasis. Meanwhile, bacterial TrxR is a small enzyme (35 KDa/subunit) free from selenium, and ebselen react with it to form Se-Se bond to act as a competitive inhibitor that could upregulate ROS production (Lu et al., 2013; Lu and Holmgren, 2014a; Zou et al., 2017).

In recent studies, we and other groups have shown that ebselen has potent antimicrobial activity against most Gram-positive bacteria including MDR *S. aureus* (Lu et al., 2013; Thangamani et al., 2015). As a well-known organo-selenium compound, ebselen is under phase II clinical trials for stroke, hearing loss, atherosclerosis, and other conditions, which guaranteed its safety and tolerance in human being (Pratta et al., 1998; Ishii et al., 2000; Zhang et al., 2002; Johnsen-Soriano et al., 2007). In this study, the bactericidal activity of ebselen was confirmed by visible spectrophotometer, transmission electron microscopy, DTNB assay, and Flow cytometry *in vitro*. These results verified the potent bactericidal activity of ebselen against MDR *S. aureus*, and demonstrated one of its ability to inhibit bacterial TrxR and disrupt the intracellular redox microenvironment.

The bacterial Trx system is important for reducing various cellular antioxidants, such as RNR, Msr, Tpx, Bcp. Thus, inhibition of TrxR is highly related to the excessive production of ROS, which may disrupt many physiological processes and cause cellular damages or even cell death (Zou et al., 2017). In order to detect the intracellular ROS level, ebselen-treated cells were stained with H_2_DCFH-DA. FACS results confirmed that ebselen inhibited the electrons transfer from TrxR to its corresponding oxidized substrates, which greatly influences ROS removal. This result demonstrated that ROS elevation is a major player in determining the bacterial fates, which is consistent with previous reports showing that ROS produced by some clinically used antibiotics contribute to their bactericidal efficacy (Kohanski et al., 2007; Morones-Ramirez et al., 2013; Zou et al., 2018).

Further, the rat model with staphylococcal skin wound infection of *S. aureus* LT-1 strain was used to determine the therapeutic efficacy of ebselen on bacterial burden and infection-induced inflammation, *in vitro*. As this model represented a staphylococcus infection of open skin wounds, it provided the opportunity to evaluate the efficacy of topical ebselen treatment. Ebselen showed high therapeutic efficacy against MDR *S. aureus* LT-1 caused skin infection, including substantially reducing the bacterial burden by day 5 (10-fold), increasing fibroblasts and microvascular, and reducing scattered lymphocytes. Moreover, since increased host inflammatory cytokines are mediators for clinical severity of staphylococcal skin infections, the expression of infection-induced inflammation cytokines was also evaluated (Montgomery et al., 2013; Sharma-Kuinkel et al., 2013). The results showed that TNF-α, IL-1β, and IL-6 all contributed to host defense during a *S. aureus* skin wound infection, whereas IL-1β was more critical during a deeper intradermal *S. aureus* infection. A similar study demonstrated that during the intradermal infection, the inducible IL-1β response of the bone-marrow-derived recruited cells of the abscess was a more critical determinant for host defense (Miller et al., 2007). Our results showed that ebselen dramatically decreased the infection-induced inflammation that provide a favorable outcome in wound healing, which might be highly related to its recognized immune-modulatory, anti-inflammatory, and anti-oxidant activities (Schewe, 1995; Ren et al., 2017, 2018). Together, the results demonstrated that ebselen has a higher skin repairing efficacy than gentamycin, which may make it a better antimicrobial than gentamycin, especially because it is predicted to have less severe side effects.

Moreover, we have investigated whether ebselen has the potential to act synergistically with the traditional polyphenol, curcumin, against *S. aureus*. The results showed that TrxR is one of the antibacterial targets of curcumin, and ebselen sensitized its ability.

Together, these results suggested that ebselen could serve as an alternative topical agent to gentamycin to treat Staphylococcal skin infections. The sensitivity of bacteria to ebselen is dependent on the antioxidant systems equipped in bacteria. Notably, ebselen increased the sensitivity of *S. aureus* to curcumin, which is a commonly used polyphenol antimicrobials component. Importantly, the combination of ebselen with other antimicrobials may help overcome the MDR in bacteria.

## Materials and Methods

### Rat and Bacterial Strains

Sprague Dawley rats (male, 180–200 g) were purchased from China Three Gorges University, and approval from the Medical Animal Care & Welfare Committee of China Three Gorges University was obtained prior to using the animals for research. All rats were kept in individually cages under a constant dark-light cycle in a conventional SPF animal house and were free access to food and water.

*Staphylococcus aureus* LT-1 was isolated from the skin lesion of a clinical patient in the First Affiliated Hospital of Three Gorges University in Hubei Province, China, with an approval for research from the Ethics Committee of the First Affiliated Hospital of Three Gorges University and written informed consent of the patient. *S. aureus* LT-1 strain was thoroughly identified by biochemical, 16S rRNA PCR (5′-GATAACCTACCTATAAGACT-3′ and 5′-TCCATCTATAAGTGACAG-3′ as primers), and MALDI-TOF MS (bioMrieux VITEK MS), and stored in our laboratory (Tables 1, 2 and Supplementary Figure S2). *S. aureus* ATCC25923 was stored in our lab.

### Regents

Bacteria cells were cultured in Luria Bertani (LB) medium (EMD millipore). 2-Phenyl-1, 2-benzisoselenazol-3(2H)-one (ebselen) (Daiichi), protease inhibitor cocktails (Roche), *Escherichia coli* DHB4 Trx was from IMCO Corp. (Stockholm, Sweden)^1^, Rabbit anti-sheep IgG-HRP (Santa cruz), Curcumin (Selleck), all the other reagents were from Sigma-Aldrich.

### The Inhibition of *S. aureus* LT-1 Growth by Ebselen

The inhibition of *S. aureus* LT-1 growth by ebselen was measured by visible spectrophotometer. *S. aureus* LT-1 cells were grown (37°C, 220 rpm) till an OD _600 nm_ of 0.4 and diluted 100 times to be treated with different concentrations of ebselen (gentamycin was used as positive control) for 16 h at 37°C in 96 wells plate, and the absorbance values at 600 nm were measured.

In addition, nuclear staining reagent propidium iodide (PI) was used to detect the inhibition efficiency of *S. aureus* LT-1 by ebselen. LT-1 cells were grown (37°C, 220 rpm) till an OD _600 nm_ of 0.4 and treated with 22 μg/ml ebselen for 40 min at 37°C, and washed three times with PBS and the cells were collected by centrifugation (6,000 rpm, 5 min). Nuclei were stained by 5 μg/ml PI for 30 min at 37°C, and were washed three times and re-suspended in PBS after the incubation, and the fluorescence measured by flow cytometry (BECKMAN COULTER, CytoFLEX).

### Effect of Ebselen on Bacterial Morphology

*Staphylococcus aureus* LT-1 was grown till an OD _600 nm_ of 0.4 in LB medium, and treated with 22 μg/ml ebselen, 64 μg/ml gentamycin and PBS for 20 min, respectively. Cells were obtained by centrifuging (4°C, 13000 rpm, 15 min), and fixed with 2.5% glutaraldehyde. The morphology and structure of *S. aureus* cells were observed under transmission electron microscopy (Hitachi H-7500, Japan).

### The Inhibition of *S. aureus* LT-1 TrxR Activity by Ebselen

*Staphylococcus aureus* LT-1 cells were cultured till an OD _600 nm_ of 0.4 and incubated with 22 μg/ml ebselen for 40 min. LT-1 cells were obtained by centrifugation (4°C, 5000 rpm, 5 min), which were washed three times and re-suspended in 50 mM Tris–EDTA buffer (pH 7.4), and the protein inhibitor cocktail was added to decrease the protease activity. Finally, the cells were disrupted with before sonication (240 W, 5 min). The cell supernatants were obtained by centrifugation (4°C, 12000 rpm, 10 min), and the protein concentration was measured by Bradford assay and used for TrxR activity assay.

The determination of TrxR activity was performed in 96 wells plate. 25 μg obtained cell lysate was incubated with 2 mM EDTA, 200 μM NADPH at 37°C for 5 min, and added 5 μM *E. coli* Trx, 2 mM DTNB. The absorbance at 412 nm was detected with a VERSA micro-wells plate reader for 5 min, and the slope was used to represent TrxR activity. The activity of the untreated group was considered as 100%.

### Determination of ROS Production in Ebselen Treated *S. aureus* LT-1

*Staphylococcus aureus* LT-1 was grown till an OD _600 nm_ of 0.4 in LB medium, and incubated with 22 μg/ml ebselen for 40 min. The LT-1 cells were obtained by centrifugation (4°C, 5000 rpm, 5 min). The pellets were washed three times with PBS and stained with 10 μM H_2_DCFH-DA for 30 min at 37°C. After the incubation, cells were washed three times and re-suspended in PBS, and the ROS production was quantified by flow cytometry (BECKMAN COULTER, CytoFLEX).

### Rat Model of *S. aureus* LT-1 Skin Wound Infection

All experiments were performed in accordance with the relevant guidelines and regulations. Healthy male SD rats (body weight 180–200 g) were used for this study. Animals were housed adaptively for 1 week in SPF room and were free access to food and water. For bacterial load and wound healing tests, thirty rats were randomly divided into three groups: control group, ebselen-treated group, and gentamycin-treated group. Thick scalpel cuts were made into the dermis of the skin on the posterior upper back and neck of rats and inoculated with 200 μl 3.26 × 10^9^ CFU/ml *S. aureus* LT-1 cells. Rats were then treated intradermal administered with 80 μl 25 mg/kg ebselen, 5 mg/kg gentamycin or PBS once per day for 5 days, respectively. Twenty-four hours after the last treatment, the wound was excised for bacteria load on *S. aureus* after homogenization. The wound healing was calculated by formula as follows: *F* = (A−B)/A^∗^100%, where F: wound healing rate, A: original wound area, and B: wound area post-treatment.

For inflammatory cytokines, pathological and IHC detection, forty rats were randomly divided into four groups: A to D, and the skin cuts were constructed as described above. Further, rats from group A served as a control group and were not infected or treated. Rats of groups B–D were inoculated with 200 μl of *S. aureus* strain LT-1 (3.26 × 10^9^). Group B was intradermal administered 80 μl PBS; Group C and group D were administered 80 μl 25 mg/kg ebselen and 5 mg/kg gentamycin, respectively. All groups were treated once a day for 5 days. Blood serum was used to detect the cytokines level by ELISA. TNF-α, IL-6, and IL-1β ELISA Kits (Uscn Life Science, Inc.) were used for the quantification of cytokines. The experiment was carried out as the manufacture instructions.

### Histological Analysis

Rats from four groups were treated as described above, and were euthanized and lesional 8-mm punch biopsy (Acuderm) tissues specimens were bisected and fixed in formalin (10%) and embedded in paraffin. Paraffin sections (4 μm thick) were cut and stained with hematoxylin and eosin and Masson stain.

Further, anti-CD64 antibody and anti-S100a4 antibody were used to present neutrophils and fibroblasts, respectively, by IHC.

### Synergistic Antibacterial Activity of Ebselen Combined With Curcumin

*Staphylococcus aureus* ATCC25923 cells were incubated till an OD_600 nm_ of 0.4 and diluted 100 times in LB medium. In 96 wells plate, the diluted bacteria were incubated with serial concentrations of ebselen and curcumin for 16 h at 37°C and read the OD_600__ nm_ values by VERSA microplate reader. In addition, PI staining was used to identify whether cells were synergistically killed by 5 μM ebselen and 80 μM curcumin as described above.

Further, the TrxR activity and ROS production were performed by DTNB and flow cytometry, respectively, to detect the synergistically inhibition effect of 5 μM ebselen and 80 μM curcumin on *S. aureus* as described above.

### The Synergist Degree Measurement

The synergist degree was measured by Bliss independent model which described as previous study and the synergist degree S was calculated by the following formula (Hegreness et al., 2008): *S* = (f_X__0_/f_00_)(f_0__Y_/f_00_)-(f_XY_/f_00_), f_XY_ refers to the growth rate of bacteria under the two drugs in combination treatment, the concentration of one drug is X and the other is Y; f_X__0_, f_0__Y_ refers to the growth rate of bacteria just one drug treatment, the concentration of one drug is X and the other is Y; f_00_ refers to the growth rate of bacteria without drug; S refers to the degree of synergy. The value of the combined degree S is between −1 and 1, and the value is closer to −1, indicating that the antagonism effect, and the value is closer to 1, indicating that the synergy efficient.

### Statistical Analyses

Statistical analyses were assessed by Graph Pad Prism 6.0 (Graph Pad Software, La Jolla, CA, United States). Statistical analysis was assessed between two groups using the Student’s two–tailed *t*-test; among multiple groups comparisons, one-way ANOVA analysis was performed. *P* < 0.05 was considered to indicate a statistically significant difference. *P*-values of < 0.05 were considered as significant (^∗∗^*P* < 0.01; ^∗∗∗^*P* < 0.001).

## Data Availability Statement

The raw data supporting the conclusions of this article will be made available by the authors, without undue reservation, to any qualified researcher.

## Ethics Statement

The animal study was reviewed and approved by the Medical Animal Care & Welfare Committee of China Three Gorges University.

## Author Contributions

CD, JZ, PW, TL, and YZ performed the experiments. JL and AH conceived the project. XR, JW, and LZ drafted the manuscript and analyzed the results.

## Conflict of Interest

The authors declare that the research was conducted in the absence of any commercial or financial relationships that could be construed as a potential conflict of interest.

## Footnotes

1 http://www.imcocorp.se

## Supplementary Material

FIGURE S1 16 S rRNA Identification of *Staphylococcus aureus* LT-1. *S. aureus* LT-1 cells was identified by 16 S rRNA PCR using primers as following: 5′-GATAACCTACCTATAAGACT-3′ and 5′-TCCATCTATAAGTGACAG-3′, and the predictive product is 115 bp. M, marker; 1–4, PCR products.

FIGURE S2 Synergistic activity of ebselen with curcumin. The Bliss Model for Synergy confirms a synergistic effect, between 5 μM enselen and 10 μM curcumin. Degree of synergy was quantified after 2 h of treatment with ebselen in combination with curcumin.
