# Supplementary figures and images for: Topical Therapeutic Efficacy of Ebselen Against Multidrug-Resistant Staphylococcus aureus LT-1 Targeting Thioredoxin Reductase
Source: Front Microbiol. 2020 Jan 15;10:3016. doi: 10.3389/fmicb.2019.03016 (PMC6974526; doi:10.3389/fmicb.2019.03016)

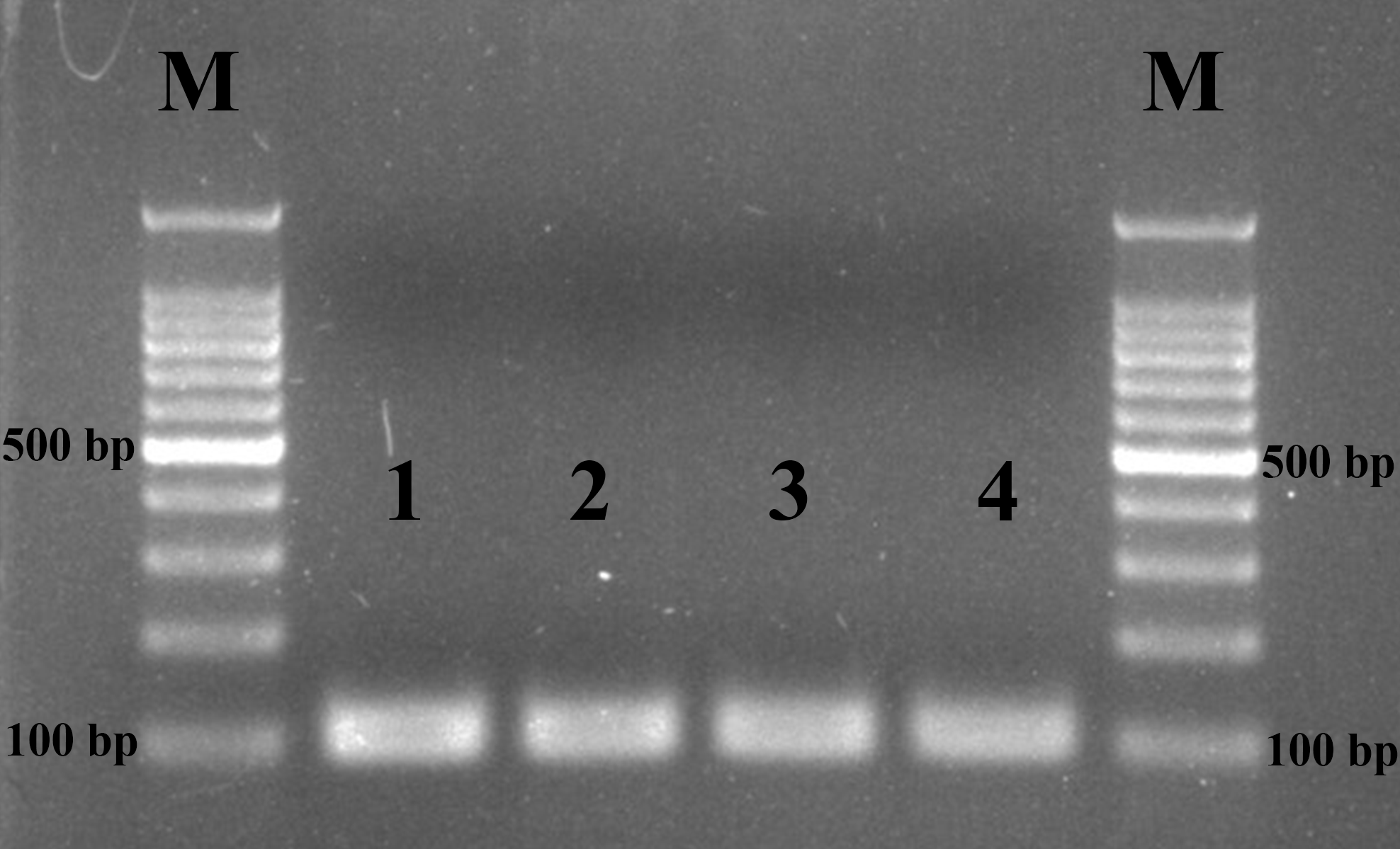

Supplement: FIGURE S1 — 16 S rRNA Identification of Staphylococcus aureus LT-1. S. aureus LT-1 cells was identified by 16 S rRNA PCR using primers as following: 5′-GATAACCTACCTATAAGACT-3′ and 5′-TCCATCTATAAGTGACAG-3′, and the predictive product is 115 bp. M, marker; 1–4, PCR products. [file Image_1.TIF]

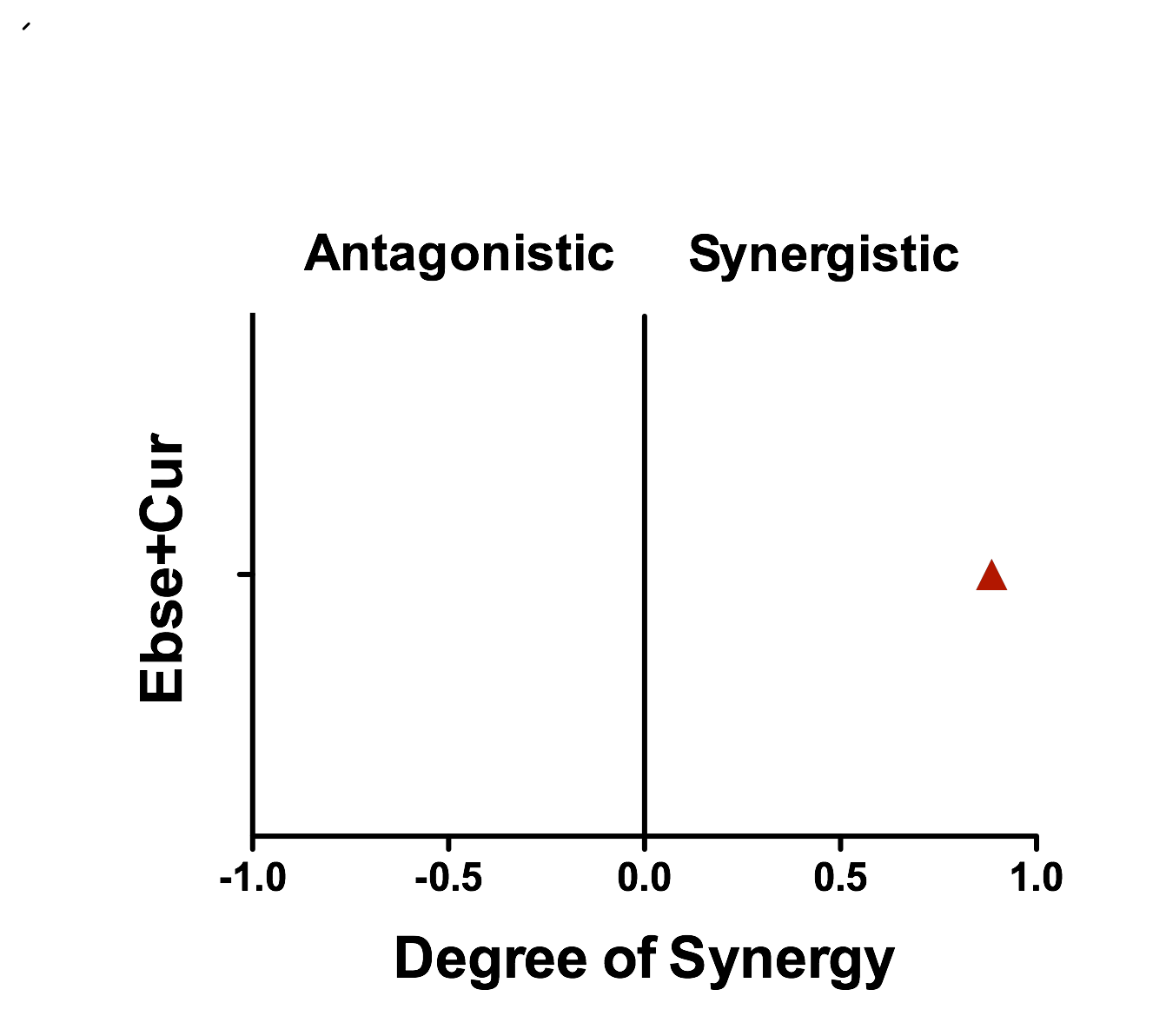

Supplement: FIGURE S2 — Synergistic activity of ebselen with curcumin. The Bliss Model for Synergy confirms a synergistic effect, between 5 μM enselen and 10 μM curcumin. Degree of synergy was quantified after 2 h of treatment with ebselen in combination with curcumin. [file Image_2.TIFF]
